# PARTNERING FOR SUCCESS: INVOLVING NURSING HOME CORPORATIONS AND SYSTEMS IN PRAGMATIC CLINICAL TRIALS

**DOI:** 10.1093/geroni/igae098.1372

**Published:** 2024-12-31

**Authors:** Christine Hartmann, Hope Carwile, Sharon Sloup, Tanya Taylor, Kathy Curci-Delgaro, Valerie Clark, Princess Nash, Liam Fry

**Affiliations:** VA Bedford Healthcare System & University of Massachusetts Lowell, Bedford, Massachusetts, United States; Vivage, Lakewood, Colorado, United States; Tuscaloosa VA Medical Center, Tuscaloosa, Alabama, United States; Department of Veterans Affairs, Washington, District of Columbia, United States; VA Medical Center, Washington, District of Columbia, United States; VA Bedford Healthcare System, Bedford, Massachusetts, United States; Tuscaloosa VA Medical Center, Tuscaloosa, Alabama, United States; The University of Texas Austin, Austin, Texas, United States

## Abstract

Partnership with nursing home organizations is critical to implement and sustain embedded interventions. We present two successful examples from ongoing nursing home embedded pragmatic clinical trials. One example is from an National Institute on Aging-funded trial of improving sleep for nursing home residents in 3 large nursing home corporations that involved a pilot followed by a trial. During the pilot phase, external consultants trained nursing home staff in the intervention and simultaneously trained a corporate “coach” to be a train-the-trainer in the trial. Engaging corporate partners informed the research team’s understanding of the context and culture and the corporate coaches’ understanding of the intervention. During the 4-year trial, occuring now, corporate coaches serve as trainers for all participating nursing homes’ staff, with limited external support. The other example is from a Department of Veterans Affairs (VA)-funded trial on preventing resident falls in State Veterans Homes. For this study, researchers partnered with an existing VA program focused on delivering a similar intervention. Because the existing VA program recently expanded into State Veterans Homes, and because research team members are expert in the intervention, partnership was a win for both parties. The existing VA program coaches gained an opportunity to work with State Homes that were eager to participate, agumented with additional support afforded by the research team. The research team gained extra help in implementing the intervention from experienced coaches. In both trial examples, partnership is pragmatic and key to the trial’s success.

